# Modeling Brownian Motion as a Timelapse of the Physical, Persistent Trajectory

**DOI:** 10.1021/acs.jpcb.4c07685

**Published:** 2025-05-07

**Authors:** Ludovico Cademartiri

**Affiliations:** Department of Chemistry, Life Sciences and Environmental Sustainability, 9370 University of Parma, Parco Area delle Scienze 17 A, Parma 43121, Italy

## Abstract

While it is very common to model diffusion as a random walk by assuming memorylessness of the trajectory and diffusive step lengths, these assumptions can lead to significant errors. This paper describes the extent to which the physical trajectory of a Brownian particle can be described by a random walk. Analysis of “timelapses” of physical trajectories (calculated over collisional time scales using a velocity autocorrelation function that captures the hydrodynamic and acoustic effects induced by the solvent) yielded two observations: (i) these subsampled trajectories become genuinely memoryless only when their time step is ∼200 times larger than the relaxation time, and (ii) the distributions of the subsampled step lengths have variances that are significantly smaller than the diffusional ones (usually by a factor of ∼2). This last observation is due to two facts: diffusional displacements are mathematically “superballistic” at short time scales, and subsampled trajectories are “moving averages” of the underlying physical trajectory. The counterintuitive result is that the mean squared displacement (MSD) of the physical trajectory asymptotically approaches 2Dt (where D is diffusivity) at long time intervals *t*, but the MSD of the individual subsampled steps does not, even when their duration is several hundred times larger than the relaxation time. I discuss how to best account for this effect in computational approaches.

## Graphical abstract

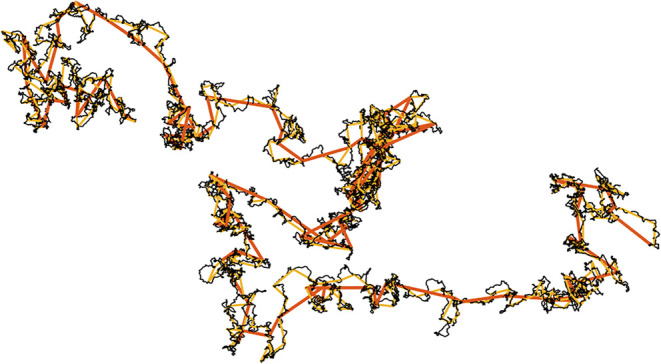

## Introduction

How does the nucleation of crystals occur? How do proteins fold and interact with ligands, surfaces, and each other? How does diffusion work in crowded environments? The dynamics of objects in liquids is the *trait d’union* of these questions of great practical and fundamental importance.

One of the challenges with studying such ensembles is that, on one hand, they are often too numerous and composed of objects too large to be conveniently described at an atomic scale;^1^ on the other hand, they are often composed of objects too small to be accurately described by most Brownian dynamics approaches.^2^ One strategy to approach this problem is to make computation at the atomic scale more efficient^3^ or to find better approximations of mean fields.^4^ Another strategy, which I espouse here, is to make Brownian descriptions more accurate and practical with smaller objects.^5^

### Time Scales

Brownian dynamics simulations (BDS) usually start from the assumptions that (i) the collisions with the solvent can be described by friction, i.e., viscosity, and (ii) the motion of the particle is not influenced by inertia (of the particle or of the solvent around it). Both of these assumptions are associated with time scales.^6^

#### Collisional Time τ_c_

The “collisional” time scale is defined here as the mean time that separates two solvent/particle “collisions”. Since there are no real collisions in liquids (nearest neighbors are always “in contact”), by “collision” I intend here any transfer of momentum between two neighboring particles (which depends on the magnitude of the orbital overlap). I estimate the mean time between solvent/particle collisions by using the following logic.

If I assume the solvent is a weakly interacting liquid with a structure close to random close packing the average number of solvent molecules in contact with the particle is $∼0.886\cdot {\left(r_{p}/r_{s}\right)}^{2}$ , where *r*_*p*_ is the radius of the particle, *r*_*s*_ is the radius of a solvent molecule, and 0.886 is the packing fraction for a random close-packed arrangement of circles on a surface.^7^

Any displacement that could trigger a transfer of momentum by “contact” must cause a sufficient overlap between atomic orbitals and therefore should be in the order of an atomic radius, i.e., ∼1 Å. For each solvent molecule on the surface of the particle, the root-mean-square (RMS) time it takes to move by 1 Å is $1\cdot 10^{-10}/v_{\mathrm{RMS},s}=1\cdot 10^{-10}\sqrt{m_{s}/{3k}_{B}T}$ , where v_RMS,s_ is the RMS velocity of the solvent molecules, k_B_ is Boltzmann constant, T is the temperature and m_s_ is the mass of a solvent molecule. Considering that during this time a solvent molecule can move toward the particle or away from it, and that the particle can also move causing a collision with 1/2 of the particles on its surface, the mean time τ_c_ between two consecutive solvent/particle collisions can be estimated (cf. Supporting Information for the step-by-step derivation) as

1 $$\tau_{c}≅\frac{9.775\cdot 10^{-11}}{\sqrt{k_{B}T}}{\left(\frac{r_{s}}{r_{p}}\right)}^{2}\frac{\sqrt{m_{s}m_{p}}}{\sqrt{m_{s}}+\sqrt{m_{p}}}$$

This time scale is most important: if one wants to describe the dissipation of momentum of the particle by the solvent/particle collisions by using viscosity rather than by describing the individual solvent/particle collisions, one needs to consider time scales τ_c_ such that the number of collisions occurring at each step is large enough to give a stable mean effect.

The largest contributor to τ_c_ is the size of the particle relative to the solvent, with the physical properties of the solvent and the temperature being minor effects (cf. Figure S1).

#### Relaxation Time τ_r_

While τ_c_ tells us at what time scales we can make a continuum approximation of the solvent’s viscous drag, the effect that each solvent/particle collision has on the dissipation of the momentum of the particle must depend on its inertia and, therefore, on its density.

In the first order approximation by which the viscous force χ acting on the particle is proportional to its velocity, the decay of particle’s momentum (and therefore of the inertial effects) occurs exponentially with a decay constant τ_r_, called the “relaxation time”,^6d^

2 $$\tau_{r}=\frac{m_{p}}{\chi };,\;\mathrm{for spheres}:\tau_{r}=\frac{2}{9}\frac{\rho_{p}}{\eta }r_{p}^{2}$$

where ρ_p_ is the particle density and η is the dynamic viscosity of the solvent. This relaxation time quantifies the time scale over which the momentum of the particle is dissipated (both in direction and magnitude) by the collisions with the solvent molecules.

These two time scales have opposite dependencies on the size of the particle: τ_c_ goes with r_p_^–2^ while τ_r_ goes with r_p_^2^. This fact implies that, depending on the solvent and on the density of the particles, there is a particle radius below which the approximations I have made so far cease to make physical sense (cf. Figure S2): physically speaking, below a certain particle size (and hence, mass) it takes very few collisions with the solvent molecules, even one, to significantly dissipate the momentum of the particle. In these conditions viscosity cannot describe dissipation, eq 2 ceases to be valid, and a full collisional description of the system (i.e., Molecular Dynamics, MD) is required. This critical particle radius (cf. Figure S2) lies in the nanoscale for the most common solvents and solid phases.

#### Random Walks as Model Systems of Brownian Motion

In most computational and theoretical studies of Brownian systems the motion of the particles is approximated as a random walk whose steps occur over fixed time intervals.^8^ Therefore, the choice of the time interval is crucial.

Beside τ_r_ and τ_c_, there is a third time scale that is often employed in BDS and chosen as the random walk time step: the time τ_e_ associated with an RMS displacement σ (usually assumed to be diffusive, i.e., $\sigma =\sqrt{6D\tau_{e}}$ , where D is diffusivity) equal to a radius of the particle (i.e., for spheres, τ_e_ = r_p_^2^/6D).

The question that remains is therefore to what degree we can ignore autocorrelations in the description of motion of the particles. If we use a random walk, each step has no correlation with any step prior in neither orientation nor displacement. On the other hand the expression for τ_r_ in eq 2 assumes the autocorrelation drops by ∼ 64% over τ_r_. At what time step does it drop to zero and we can safely use a random walk? Is τ_e_ a long enough time interval?

### Dealing with Autocorrelation, Memory, Persistence

Particles in solution possess inertia. This inertia causes their momentum to persist, only to slowly decay over time due to the collisions with the solvent. This means that the velocity ***v*** of a particle at time *t* is not necessarily independent from the velocity of the same particle at time *t* – λ where λ is called the “lag”: the motion is said to be “autocorrelated”. This correlation is quantified (as a function of the lag λ) by the velocity autocorrelation function (VACF) and expressed as

3 $$C_{v}\left(\lambda \right)=\langle v\left(t\right)\cdot v\left(t+\lambda \right)\rangle$$

where the angle brackets ⟨···⟩ denote an ensemble average over all particles and over different time origins *t*.

What the VACF quantifies can be described usefully in several ways: it quantifies (i) how the momentum of the particle fades with time, (ii) the autocorrelation of the velocity, (iii) the memory of the trajectory, and (iv) the persistence of motion. These are all different terminologies that are used in different scientific communities to describe the same thing. As we will see later on, the VACF is where the physics of the system is described.

The advantage of the VACF formalism is that, once the VACF is known, the dynamics of the system can be derived from it. For example, the diffusivity can be determined using Green–Kubo relation $D=\int_{0}^{\infty }C_{v}\left(\lambda \right)d\lambda$ , and the MSD in one coordinate (MSD_*x*_, σ_*x*_^2^) can be determined^9^ using

4 $$\sigma_{x}^{2}\left(t\right)=2\int_{0}^{t}(t-\lambda )C_{v}\left(\lambda \right)d\lambda$$

It is easy to calculate *C*_*v*_ if one has access to a large set of time series: i.e., if I measure with a microscope the trajectories in time of a large number of Brownian particles I can easily calculate the VACF of the ensemble using eq 3. The inverse problem—creating *ex novo* a time series whose autocorrelation is *C*_*v*_—is possible but much more complicated. In the case of a time series of velocities, I would write

$$v_{x}\left(t\right)=\overset{\lambda_{max}}{\underset{\lambda =1}{\sum }}\beta_{\lambda }v_{x}\left(t-\lambda \right)+\xi \left(t\right)$$ 5

where v_*x*_(t) is the time series whose autocorrelation is supposed to be *C*_*v*_. eq 5 tells the following story: the velocity v_*x*_ at time *t* is just the *weighted* average of the velocities at prior times *t – λ* with λ that goes from 1 to a maximum lag λ_max_ plus a random function ξ (generally normally distributed with a known variance). Therefore, the weights β_λ_ (so-called “autoregression coefficients”), if they could be determined, would allow one to calculate any number of time series from scratch knowing that they would obey the autocorrelation function *C*_*v*_ and the underlying physics.

The autoregression coefficients can be calculated from the VACF by solving the corresponding Yule-Walker system of equations

6 $$\left[\begin{array}{l} C_{v,1}\\ C_{v,2}\\ C_{v,3}\\ \vdots \\ C_{v,\lambda_{\max}} \end{array}\right]=\left[\begin{array}{llll} C_{v,0} & C_{v,1} & C_{v,2} & \mathrm{...}\\ C_{v,1} & C_{v,0} & C_{v,1} & \mathrm{...}\\ C_{v,2} & C_{v,1} & C_{v,0} & \mathrm{...}\\ \vdots & \vdots & \vdots & \ddots \\ C_{v,\lambda_{\max}\mathrm{-1}} & C_{v,\lambda_{\max}\mathrm{-2}} & C_{v,\lambda_{\max}\mathrm{-3}} & \mathrm{...} \end{array}\right]\left[\begin{array}{l} \beta_{1}\\ \beta_{2}\\ \beta_{3}\\ \vdots \\ \beta_{\lambda_{\max}} \end{array}\right]$$

where the left side is the vector of the autocorrelation function *C*_*v*_ (numerical subscripts indicating the lag), while the right side is the product of the Toeplitz matrix of *C*_*v*_ (from λ = 0) and the vector of the autoregression coefficients.^10^

That might look easy but is not for the following reasons: (i) if the time steps are small, λ_max_ has to be large, and eq 6 can become computationally intractable; (ii) if the autocorrelation vanishes slowly over time (e.g., with a long, algebraic tail) it is difficult to choose λ_max_ (the tail can have a disproportionate effect on the behavior of the system even though the absolute values of autocorrelation there are very small); (iii) the generated time series *v*_*x*_(*t*) can be extraordinarily sensitive to the accuracy of the β coefficients and therefore to the accuracy of the VACF (and therefore its numerical integration) and to the appropriate truncation (e.g., choosing a excessively small λ_max_ can cause significant deviations between the generated time series and the original ones).

How does this whole discussion relates to the problem of Brownian dynamics? BDS generate trajectories *ex novo* that should obey a specific VACF. In theory then BDS should use eq 5, but in practice they usually assume β_λ_ = 0 by choosing time steps in the simulation for which the VACF, in theory, should be negligible. In simpler terms, they assume the motion is uncorrelated, memoryless, nonpersistent, i.e., a random walk. My goal with this work was to test the limits of this assumption.

## Computational Design

### Assumptions and Terminology

In what follows I only consider spherical objects in the absence of advective flows. Nonsphericity and shear introduce significant complications in the description of motions, collisions, and viscosity.^11^ For convenience I also describe the distinct objects that make up the liquid as “solvent molecules” (even though there is no requirement for them to be molecules), and the object being moved by collisions with the solvent molecules as the “particle” (even though there is no requirement for it to be a solid phase and not a molecule).

### Testing Validity of Random Walks as Models for Physical Brownian Trajectories

My approach is as follows (Figure 1 summarizes the entire approach in detail; we reference this figure repeatedly as we progressively clarify in detail the individual steps). (step 1) I calculate what I call here the “physical trajectory”: a trajectory that should be as close as possible to the real trajectory performed by a particle in solution (in other terms, the so-called “ground truth” of the model). The strategy I use to calculate it is explained in the discussion later on. (step 2) While calculating these physical trajectories, I compile timelapses, i.e., I record the position of the particles at time intervals that are greater than the time interval used to calculate the physical trajectory. The compiled timelapse is the same trajectory, i.e., still physically correct, but sampled at larger time intervals. By analogy, if the “physical trajectory” is a movie shot at 30 frames per second, the timelapse is the same movie compiled by taking one every three frames. (step 3) These timelapses can now be analyzed from scratch as if they would be new data (cf. Figure 1D): their MSD and their VACF can be calculated (we will discuss later why this VACF is not just the “timelapse” of the VACF we used to calculate the “physical trajectory”) and from them the autoregression coefficients can be extracted. (step 4) The result of this procedure is that we have now autoregression coefficients that allow us to create trajectories ex novo that reproduce correctly the autocorrelation originating from the physical trajectory but without having to use extremely small time steps. These trajectories can now be compared to random walks to test under what conditions the random walk approximation that is nearly universally used in BDS is physically accurate.

**Figure 1:**
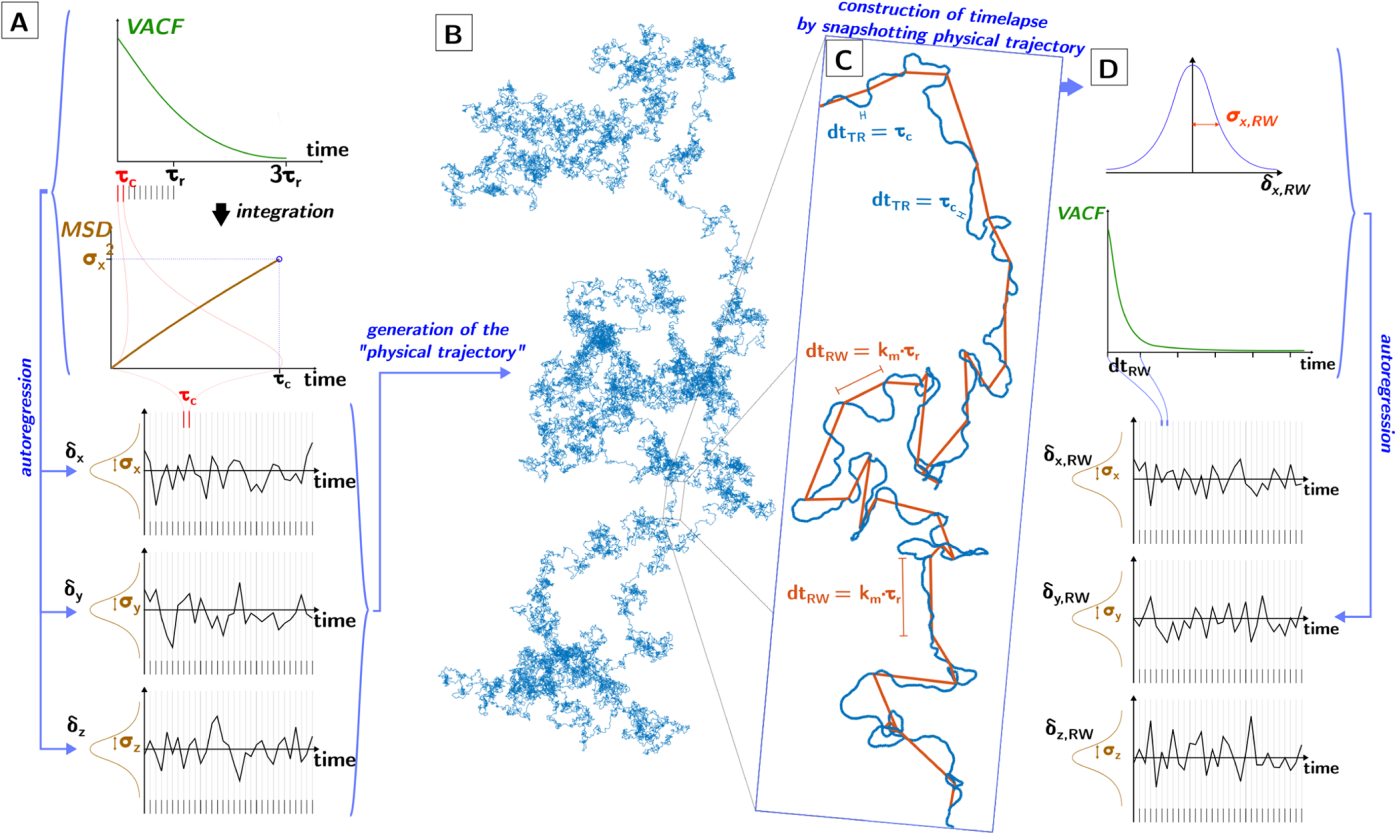
Timelapse of the physical trajectory. (A,B) *Generation of the physical trajectory.* From the top down, VACF is calculated up to 3·τ_r_ on τ_c_ intervals. The MSD at τ_c_ is calculated from a finely integrated VACF from 0 to τ_c_. The autoregression (AR) coefficients of the long-term VACF, together with the MSD at τ_c_ are used to generate autocorrelated time series of the Cartesian components of the displacement δ_*x*_, δ_*y*_, δ_*z*_ at τ_c_ intervals. (B,C) *Construction of the timelapse.* As the physical trajectory is generated (blue line), snapshots are taken at time intervals *τ*_*K*_ integer multiples of *τ*_*r*_. The assembled timelapses constitute a segmented path (orange line). (C,D) *Analysis of timelapse.* The timelapse is analyzed as a persistent random walk, extracting the MSD and VACF. From those, the autoregression coefficients are finally obtained which allow for the rapid generation ex novo of persistent random walks that accurately match the physical trajectory.

#### Simulation Parameter Space

Our simulations were conducted considering a library of relevant solvents (H_2_O, EtOH, toluene, tetrachloroethylene, and hexadecane) and particle phases (proteins, amorphous SiO_2_, calcite, Fe_3_O_4_, CdSe, and Au) and particle radii *r*_*p*_ between 1.66·10^–10^ m and 1·10^–8^ m in 10 logarithmically distributed steps.

Out of all possible combinations of these parameters, I excluded from consideration those for which m_s_ > m_p_ or τ_r_ < 10·τ_c_. This last condition ensures that at least 10 solvent/particle collisions are needed to decorrelate the motion of the particle from 1 to ∼1/e. Translating this condition in an inequality between the properties of particle and of the solvent molecules the condition leads to (cf. Supporting Information for derivation)

7 $$\rho_{p}r_{p}^{4}>\frac{3.888\cdot 10^{-9}\eta r_{s}^{2}}{v_{RMS,s}}=\Gamma_{K}$$

The right side of the inequality is a temperature-dependent property of the solvent (Γ_K_, in [kg·m]) that can be easily tabulated for reference (cf. Table 1).

**Table 1:** Sample Table of Γ_K_ Values for Different Solvents and Temperatures

Solvent	Temperature [C]	Γ_K_ [10^–33^ kg·m]
water	25	0.148
	70	0.134
toluene	25	0.692
EtOH	25	0.627
tetrachloroethylene	25	1.33
hexadecane	25	11.527
	300	8.137

Using these values it is easy to estimate the smallest particle radius r_B_ that could be reasonably described by a Brownian simulation (with the possible caveat of having to consider autocorrelation effects). For a SiO_2_ particle in water r_B_ is 0.505 nm, for CdSe in hexadecane at 300 C (close to synthesis conditions for colloidal quantum dots) r_B_ is 1.09 nm, while for Au particles in water is 0.296 nm. Importantly, for a protein in water at room temperature (considering a molecular-weight independent density of 1220 kg/m3 ^12^ r_B_ is ∼0.59 nm, smaller than most proteins.

Figure 2A shows r_B_ as a function of ρ_p_ at 298 K. The data for the plot were calculated accounting for the variation of η (through Andrade^13^ and Vogel^14^ equations using parameters from Reid, Prausnitz and Poling^15^) and ρ_s_ (through Hankinson-Brobst-Thomson technique^16^ using parameters also from Reid, Prausnitz and Poling^15^) with T. The script is included in the Supporting Information.

**Figure 2:**
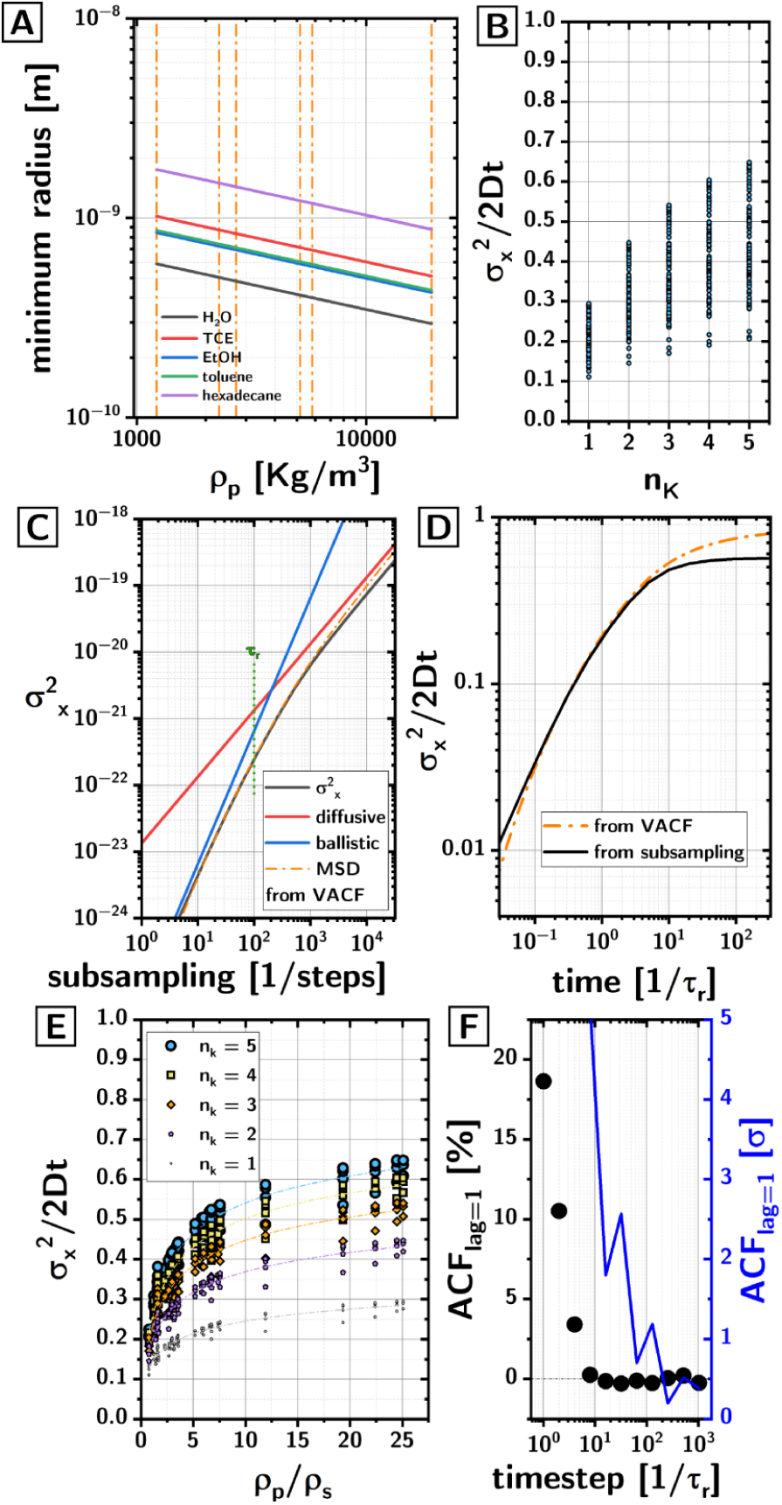
(A) Minimum radius of particles describable as Brownian (i.e., τ_r_ > 10τ_c_), as a function of their density, their phase, and the solvent. (B) Ratio between the observed variance of the step length σ_*x*_^2^ (single Cartesian coordinate) in the timelapse and the diffusional prediction 2Dt. The ratio is plotted as a function of the time resolution of the timelapse (physical trajectory is sampled at τ_k_ = n_k_·τ_r_ time intervals). (C) Variance of the step length σ_*x*_^2^ from our timelapse (black line), compared to the diffusive MSD (red), ballistic MSD (blue), and the MSD obtained from the VACF of the physical trajectory (orange dash dot). The data is plotted as a function of the time interval. The MSD obtained from the VACF transitions from ballistic to diffusive regimes around the relaxation time τ_r_, but the variance of the step length σ_*x*_^2^ from the timelapse remains offset from the diffusional prediction at long times. (D) Same data as panel C but plotted in terms of the ratio between the MSD and the diffusional prediction. (E) Ratio between the MSD and the diffusional prediction as a function of the density ratio (ρ_p_/ρ_s_) and the subsampling rate. (F) Decorrelation at lag = 1 as a function of the time step in the timelapse (in units of τ_r_), expressed either as percentage of autocorrelation (black scatters) or as standard deviations from the mean autocorrelation at large lags (blue).

Brownian dynamics *can*, in principle, be used to describe the motion in liquids of nearly everything but small molecules, as long as autocorrelation is accounted for.

## Generating and Analyzing the “Physical” Trajectory

Simulations are discrete by their own very nature. The smallest “discontinuities” in the motion of the particles in solution are the individual collisions with the solvent molecules. This means that choosing *τ*_*c*_ as the discrete time step used in calculating this “physical” trajectory seems reasonable (cf. Figure 1A). As previously explained, once the time step is determined, knowledge of the VACF should allow one to calculate the trajectory since the VACF should capture the relevant physics behind the dynamics of the particle.

### Defining the Properties of the Physical Trajectory

As we have seen from eq 4 the MSD can be determined from the VACF. The VACF decays exponentially only if caused by random noise, or, in other words, if the only source of autocorrelation is the inertia of the particle. In reality, the solvent introduces further correlation.^17^

First, as the particle moves into the liquid, it “writes” information about its own passage into the dynamics of the liquid itself: vortices (i.e., a collective, correlated motion of the liquid molecules) are created in the wake of the particle. A particle moving inside this correlated velocity field is inevitably correlated with its own past motion.^18^ The correlation vanishes in time as these vortices propagate away from the particle over the “vorticity diffusion time”, $\tau_{v}=\frac{\rho_{s}}{\eta }r_{p}^{2}$ , where ρ*_s_* is the density of the liquid.

The second effect is due to the compressibility of the liquid, which causes it to undergo compression when the particle pushes against it. This compression establishes acoustic waves that affect the velocity field of the liquid surrounding the particle at later times. The time scale of this process is the sonic time $\tau_{s}=\frac{r_{p}}{c}$ , where *c* is the speed of sound in the liquid.

These two effects are usually somewhat separated in time scale (typically *τ*_*s*_ < *τ*_*v*_, cf. Figure S3A), so they are accounted for by a VACF composed of two terms that dominate respectively at long and short time scales^17,19a^

8 $$C_{v}\left(t\right)=\frac{k_{B}T}{M}\left\{\frac{{2\rho }_{p}}{{9\pi \rho }_{s}}\int_{0}^{\infty }\frac{\sqrt{x}e^{\mathrm{-xt}/\tau_{v}}}{{1+\sigma }_{1}{+\sigma }_{2}x^{2}}\mathrm{dx}+\frac{e^{\alpha_{1}t/\tau_{s}}}{{1+2\rho }_{p}/\rho_{s}}\left[\cos\left(\frac{\alpha_{2}t}{\tau_{s}}\right)-\frac{\alpha_{1}}{\alpha_{2}}\sin\left(\frac{\alpha_{2}t}{\tau_{s}}\right)\right]\right\}$$

where $\sigma_{1}=\frac{1}{9}\left({7-4\rho }_{p}/\rho_{s}\right)$ , $\sigma_{2}=\frac{1}{81}{\left({1+2\rho }_{p}/\rho_{s}\right)}^{2}$ , $\alpha_{1}{=1+\rho }_{s}/{2\rho }_{p}$ , $\alpha_{2}=\sqrt{1-{\left(\rho_{s}/{2\rho }_{p}\right)}^{2}}$ , and *M* is the “effective mass” of the particle $M=\frac{4}{3}{\pi r}_{p}^{3}\left(\rho_{p}+\frac{\rho_{s}}{2}\right)$ : a particle moving a liquid must displace liquid to do so which means that its mass must include the mass of the displaced liquid. Upon introducing these correlation effects due to the solvent the VACF does not decay exponentially but rather with a power law tail with an exponent equal to −3/2.^18,20^

To generate the physical trajectory, I calculated this VACF for each combination of solvent and particle up to times equal to 3·*τ*_*r*_ in time intervals equal to *τ*_*c*_. The MSD, *σ*_*x*_^2^, at the time *τ*_*c*_ (i.e., the time step chosen for the physical trajectory) was calculated from the same VACF, but integrated with a much finer time resolution (*τ*_*c*_/10^4^) given the sensitivity of the integral in eq 4 to the discretization (cf. Figure 1A). The calculated VACF follows the expected power law tail with a −3/2 exponent (Figure S3B).^20^

### Calculating Ex Novo Physical Trajectories

A more delicate process is the determination of the displacements for each time step, since these have to be autocorrelated according to the *C*_*v*_(*t*) I just described. To do so, it is necessary to determine the autoregression coefficients *β*_*λ*_ for the expansion in eq 5, where the maximum lag being considered is 3·*τ*_*r*_/*τ*_*c*_. The coefficients were calculated by solving the corresponding Yule-Walker system of equations (eq 6) using a normalized VACF^10^

9 $$C^{\prime }\left(\lambda \right)=\frac{C_{v}\left(\lambda \right)}{C_{v}\left(0\right)}$$

Since many of our simulations involve lags in excess of 10^5^ timesteps, solving the system of equations in double precision can be cumbersome (since I am modeling one particle at a time, the matrices are large but I could not make processing in graphical processing units in double precision advantageous). We used Durbin-Levinson approaches to speed up the determination of the coefficients.^21^ Using this approach, the VACF of the physical trajectory produced using the autoregression coefficients as described above match closely the input VACF (cf. Figure S3C).

Once I determined the *β*_*λ*_ coefficients for the physical trajectory, the individual components of the individual displacements for each time step for each trajectory were generated as a timeseries of unitary variance by scaling the noise term ξ­(*t*) by a factor $\sigma_{n}=\sqrt{1-C^{\prime }\cdot \beta }$ , where the dot indicates a dot product of the *C’* and β vectors. The resulting time series of the individual components of the 3D displacement shows the expected variance and distribution (cf. Figure S3D). The time series is then scaled to obtain the variance consistent with *σ*_*x*_^2^ (cf. Figure 1A).

This process is executed for each spatial component, obtaining independent timeseries for the individual components of the displacement (cf. Figure 1A,B), which are then assembled as displacement vectors, and their cumulative sum is the final trajectory.

The distribution of the lengths of the 3D displacement vectors follows expectedly a Maxwell–Boltzmann distribution (cf. Figure S3E). The RMS velocities obtained from these distributions are close to the ballistic values as expected from the fact that the time step of the physical trajectory is τ_c_ (cf. Figure S3F). The difference from the ballistic values depends on the relative solvent/particle compositions and especially on their density ratio as it determines the impact of each solvent/particle collision on decorrelation. Lastly, the orientations of the displacements that make up the physical trajectory, sampled over a long enough time, appear to be ergodic (cf. Figure S3G).

The autocorrelation of the *orientation* of the displacement vectors is different than the autocorrelation in their *moduli* (cf. Figure S3H for an example). The autocorrelation of the moduli of the displacement vectors is lower than that for the individual components, while the autocorrelation of the orientation of the displacement vectors is also lower than that for the Cartesian components of the displacements but higher than for the moduli. In other words, the orientation of motion is more strongly autocorrelated at short times than the lengths of the steps.

### Subsampling the Physical Trajectories into a Timelapse

While calculating the physical trajectory (cf. Figure 1B for an example of a small section of a calculated trajectory), I took “snapshots” of the particle positions at intervals of time *τ*_*K*_ where *τ*_*K*_ = *n*_*K*_ · *τ*_*r*_, where *n*_*K*_ is an integer I call the “Kuhn multiplier” (in our simulations *τ*_*K*_ was taken between 1 and 5). The name “Kuhn multiplier” is an homage to Prof. Werner Kuhn who applied a reasoning similar to this to describe the conformation of polymers as random walks with the goal of calculating their conformational entropy.^22^

For such extracted timelapses to be then separately reproduced *ex novo* as a coarse-grained model of a physical Brownian trajectory, they should be then characterized for: (i) their step length distribution; (ii) their autocorrelation and autoregression coefficients. In what follows I describe how these functions change as a function of solvent, material and size of the particle, as well as *n*_*K*_.

## Analysis of the Timelapses

It could be tempting to think that the MSD, VACF and other quantities associated with the timelapses would be just the values of MSD and VACF from the physical trajectories at longer times. This is not the case. Subsampling changes the autocorrelation of a timeseries in often unpredictable ways, even if the overall conformation of the trajectory is preserved.^23^ This can be understood in terms of Fourier transforms: a subsampling is a low-pass filter that “forgets” high frequency correlations in a way that depends subtly on the structure of the VACF (which is why I could not just calculate the MSD at *t* = τ_K_ using the VACF of the physical trajectory).

The distributions of the single component displacements in the timelapse were exceedingly well fitted with normal distributions (R^2^ across all our conditions was 0.9993 ± 0.0001), and the displacement step acceptably well described by Maxwell–Boltzmann distributions (R^2^ = 0.998 ± 0.01).

There are five noteworthy observations.

### The Steps of the Timelapse are Subdiffusive

The first is that the steps occurring in the timelapse are shorter than what expected from diffusion even when the snapshots are taken every 5τ_r_ (cf. Figure 2B). The ratio $\alpha =\sigma_{x}^{2}/2Dt$ between the variances in step length in the subsampled trajectories and the diffusive prediction (averaged across all our simulations) was 0.44 when n_k_ = 5, i.e., when motion is often regarded as diffusive. This is not necessarily surprising for at least two reasons: (i) at very short time scales even ballistic motion is, mathematically speaking, “subdiffusive” (cf. Figure S3F showing diffusive vs ballistic MSD as a function time) and (ii) σ_*x*_^2^ of a subsampled time series are basically moving averages with a window as wide as the time interval, hence they “lag behind” the continuous time series they derive from.

At very short times, the ballistic MSD is lower than the diffusive MSD because the diffusive formula incorrectly assumes that diffusion begins at *t* = 0. In reality, diffusion only starts well after the relaxation time τ_r_; before that, the motion is partially ballistic and slower than the diffusive prediction (cf. Figure S3F). Consequently, while the diffusive MSD slightly overestimates displacement at long times (with the relative error diminishing over large intervals), using it to calculate step displacements for a random walk can introduce large errors.

It is easy to show how, for the same original physical trajectory, the σ_*x*_^2^ associated with a single time step depends on the subsampling. A single physical trajectory was calculated for 10^9^ steps and where τ_r_ ≅ 100τ_c_. The trajectory was then subsampled every 2, 4, 8 steps and so on, up to 32 768 steps. The variance of the displacements (i.e., the σ_*x*_^2^) in these subsamplings can be then compared with the diffusive and ballistic MSD obtaining the plot in Figure 2C. Importantly, the dash-dot orange curve in Figure 2C shows the MSD one could have calculated directly from the VACF of the physical trajectory at the corresponding time intervals. The discrepancy between the MSD from the subsampling and that from the VACF becomes very significant for subsamplings larger than τ_r._

Furthermore, the ratio α is different from what one would obtain directly from the VACF in eq 8 (cf. Figure S2D). The σ_*x*_^2^ derived directly from the VACF tends asymptotically to 2Dt as t increases, while the one obtained from the subsampled trajectories plateaus to a value that is significantly lower than 2Dt.

This means that picking a time interval for a BDS that is orders of magnitude larger than τ_r_ does not lead to a diffusive step length (i.e., variance σ^2^ = 6Dt) as it is usually assumed on the basis of the relationship between VACF and MSD! Making that approximation causes a *very* significant overestimate of the step lengths of the random walk. Correcting this problem is procedurally easy because σ_*x*_^2^ is still linear in *t* which means correcting the value of diffusivity will lead to the correct distribution of step lengths: if α=0.44 then one can replace D with an effective D_eff_ = 0.44·D.

### The Degree of Subdiffusion is Only Partially Predictable

The second noteworthy thing is the large spread in the values of α for identical values of n_k_. My initial hypotheses to explain this unpredictability were (i) insufficient statistics on the simulations, (ii) excessively coarse integration of the VACF or premature truncation of it, or (iii) something intrinsic to the model.

The first two hypotheses were easily disproven. First, σ_*x*_^2^ calculated from 1000 replicate trajectories gave relative standard errors on the mean of 10^–4^: the values are reproducible. Second, extending the calculation of the VACF to 100τ_r_ and decreasing the size of the *dx* interval in eq 8 by 3 orders of magnitude did not change the σ_*x*_^2^ significantly (certainly less than 1%): it is not a problem with the numerical integration or with the truncation.

The explanation for the unpredictability is hinted at by the fact that α appears to be correlated with the density ratio ρ_p_/ρ_s_ (cf. Figure 2E), without explaining fully the spread in the data. The density ratio is featured prominently in eq 8 which quantifies the VACF. The VACF in eq 8, as already stated, does not decay exponentially. Nonetheless, the τ_r_ on which I base the subsampling intervals is obtained from eq 2, which instead assumes an exponential decay of the VACF. Furthermore, the subsampling intervals I picked in order to allow for the study of small Brownian particles, are in a range where α is still rapidly changing (cf. Figure 2D). Taken together, these facts indicate that the unpredictability of the values in Figure 2B are the result of (i) the difficulty of quantifying a relaxation time from a VACF that has an algebraic decay (i.e., τ_r_ corresponds to different degrees of decorrelation depending on the density ratio and other physical variables used in eq 8), (ii) the steep dependence of α for subsampling intervals of small multiples of τ_r_.

An unfortunate consequence of this unpredictability is that the correction to apply to the diffusivity for the subsampled random walk might have to be calculated for each system one considers.

### Random Walk Approximation Requires Timesteps > 200τ_r_

Using the correct σ_*x*_^2^ for the time step chosen can be sufficient in generating accurate *ex novo* trajectories. To assess how quickly the autocorrelation vanishes as a function of the subsampling we calculated the autocorrelation of the 1D components of the displacements as a function of the subsampling in units of τ_r_ (for the calculation we used the 10^9^ step physical trajectory mentioned above). This analysis (cf. Figure 2F) shows that autocorrelation at λ = 1 vanishes for subsamplings where the time step is approximately equal to 32τ_r_. If one looks at how the autocorrelation at λ = 1 compares to the standard deviation of the autocorrelation at large λ (cf. Figure 2F, blue) then one might want to push to 200τ_r_ to ensure complete loss of autocorrelation.

### Random Walks with Steps Commensurate with Particle Radius are Generally Memoryless but Still Subdiffusive

Somewhat surprisingly, in most cases for which τ_r_ > 10τ_c_ (i.e., Brownian regime) the condition for memorylessness of the path (time step = 200 τ_r_) leads to RMS step lengths safely shorter than the diameter of the particles (cf. Figure 3A–E). In other words, a BDS whose time step is commensurate to the time it takes the particle to move by its radius can safely ignore autocorrelation. It is obviously important to keep the Brownian step significantly smaller than the particle radius since the steps are normally distributed and Brownian simulations involve often large numbers of steps: steps that are 5 or more standard deviations away from the mean cannot be excluded. This fact, especially in the case of highly concentrated systems, can cause, for example, issues in the accurate detection of collisions.

**Figure 3:**
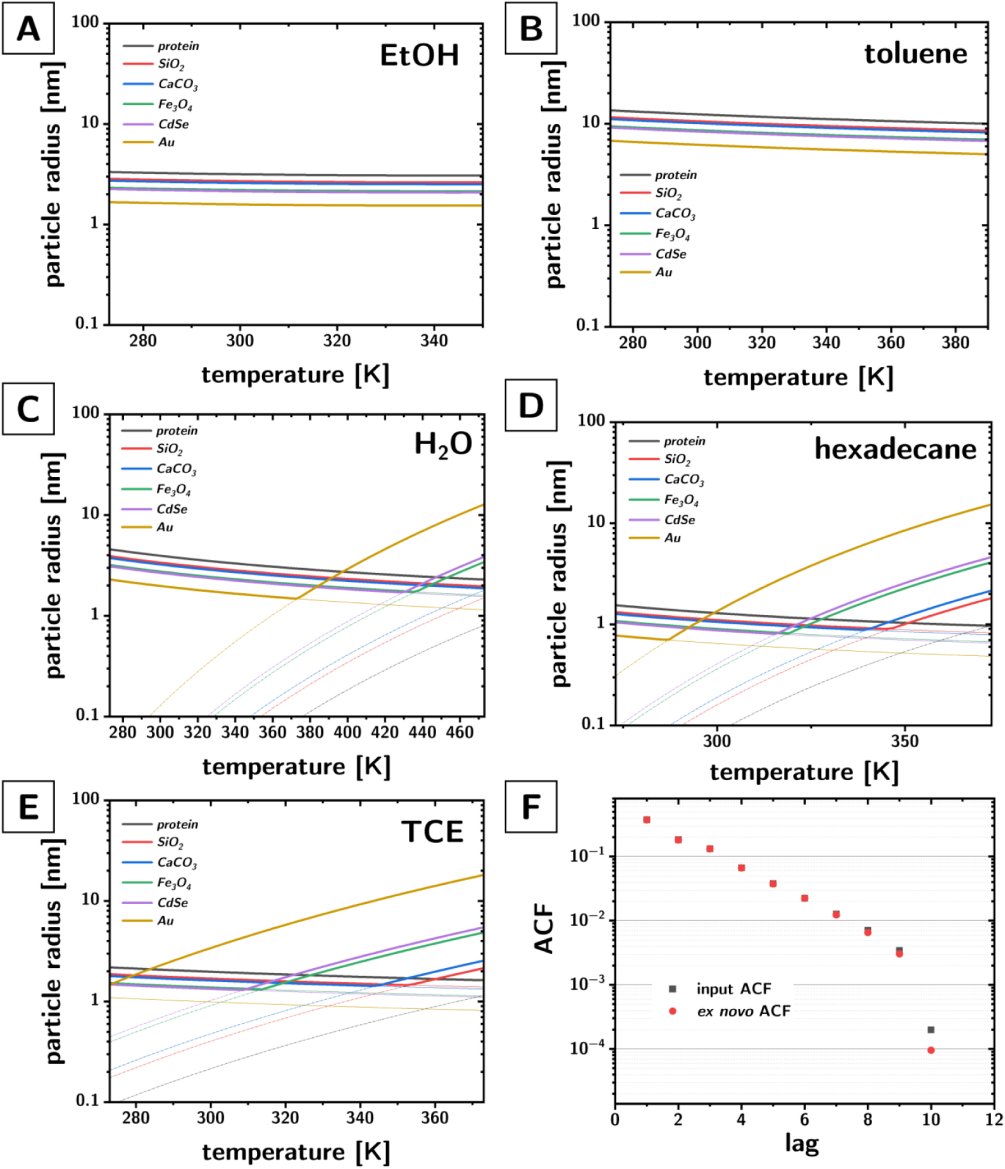
(A–E) Minimum particle radii (as a function of particle phase, temperature, and solvent) to satisfy the Brownian regime (thin colored solid lines) and the memorylessness approximation (dash-dotted thin lines), i.e., timesteps long enough to eliminate autocorrelation (τ_k_ ∼ 200τ_r_) produce RMS displacements significantly shorter than the diameter of the particles. (F) Representative comparison between the autocorrelation (ACF) obtained from the input trajectory (i.e., the timelapse of the physical trajectory), and from the ex-novo trajectories obtained with the autoregression coefficients extracted from the input ACF.

### Memory, If Necessary, can be Introduced in Random Walks to Accurately Mimic the Physical Trajectory

In the case in which instead the time step chosen is smaller than 32τ_r_, then autocorrelation must be considered. To do so, we took the subsampled trajectories, extracted their VACF, calculated the autoregression coefficients by solving eq 6, and then used eq 5 to calculate ex novo new trajectories. The noise term was normalized to give a variance equal to the σ_*x*_^2^ extracted from the original subsampled trajectory.

The results of this approach are positive. The VACF obtained from these ex novo trajectories match very closely the VACF of the original subsampled trajectory (cf. Figure 3F for a representative example).

## Conclusions

In conclusion, I have explored and discussed to what extent a Brownian trajectory can be modeled as a random walk. Diffusion is often modeled (especially in simulations of ensembles of particles) as a random walk assuming memoryless trajectories and diffusional step lengths (σ_*x*_ = (2Dt)^1/2^) on top of which corrections are then made to account for interactions. As I show here, the first approximation can be often reasonable, but the second is fundamentally wrong and leads to a very significant overestimation of the step lengths.

To find this, I have (i) calculated a physical Brownian trajectory by considering collisional time scales and a VACF that accounts for hydrodynamic and acoustic effects, (ii) taken snapshots of particle positions at time intervals that are multiples of the relaxation time, (iii) analyzed the subsampled trajectory as an autocorrelated random walk extracting MSD and VACF.

The simulations (considering a variety of solvents, particle phases, and particle sizes) show that (i) subsampled walks become genuinely memoryless only when the time step is ∼200 times larger than the relaxation time, and (ii) the distribution of their step lengths is nicely Gaussian (on each Cartesian component) but with variances that remain significantly smaller than 2Dt. This last fact is due to the combination of two effects: diffusional MSD is mathematically superballistic at short time scales and subsampled trajectories are moving averages of the underlying physical trajectory. In other words, maybe counterintuitively, the MSD of the physical trajectory at long time intervals asymptotically approaches 2Dt, but the MSD of the subsampled steps does not, even when the associated time interval is several hundred times larger than the relaxation time.

Practically speaking, given the inaccuracies of the estimated relaxation times, the appropriate corrections to the step lengths (or, alternatively, to the diffusivities) must be extracted from a simulated physical trajectory, using the physical parameters of the system at hand and the chosen subsampling. The simulations discussed here in fact show that the precise value of the correction depends intimately on the specific physical parameters (densities, radii, etc.) in ways that are not accurately captured by empirical fits.

## Supplementary Material



## Notes

Appendix I detailing the derivation for the expressions of τ_c_ and Γ_K_; Figures S1–S3 showing (i) τ_c_ as a function of solvent/particle characteristics as well as temperature; (ii) τ_r_/τ_c_ as a function of particle radii, phases and solvents; (iii) computational diagnostics (PDF)
