# Supplementary material for: Modeling Brownian Motion as a Timelapse of the Physical, Persistent Trajectory
Source: J Phys Chem B. 2025 May 7;129(22):5511–9. doi: 10.1021/acs.jpcb.4c07685 (PMC12147200; doi:10.1021/acs.jpcb.4c07685)
Supplement: Supplementary file 1 [file jp4c07685_si_001.pdf]

MODELING BROWNIAN MOTION AS A TIMELAPSE OF THE PHYSICAL, PERSISTENT, TRAJECTORY

LUDOVICO CADEMARTIRI[†*]

[†] *Department of Chemistry, Life Sciences and Environmental Sustainability, University of Parma, Parco Area delle Scienze 17 A, Parma, Italy*

[*] *Author to whom correspondence should be addressed: ludovico.cademartiri@unipr.it*

SUPPORTING INFORMATION

Appendix I

Derivation of the expression for $\boldsymbol{\tau_c}$

$$
\begin{cases}
A_p = 4\pi r_p^2; \text{ particle surface area} \\
CS_s = \pi r_s^2; \quad \text{cross-sectional area of solvent molecule} \\
N_s \sim \varphi_{rcp} \dfrac{A_p}{CS_s}; \text{ number of solvent molecules that can touch the particle} \\
\varphi_{rcp} = 0.886; \quad \text{random close packing fraction of circles in 2D} \\
dx \cong 1\text{Å}; \text{ typical displacement causing collision} \\
dt_s = \dfrac{dx}{v_{RMS,s}}; \quad \text{time associated with dx displacement by solvent} \\
dt_p = \dfrac{dx}{v_{RMS,p}}; \quad \text{time associated with dx displacement by particle} \\
v_{RMS,s} = \sqrt{\dfrac{3k_BT}{m_s}}; \quad \text{RMS velocity of solvent molecule} \\
v_{RMS,p} = \sqrt{\dfrac{3k_BT}{m_p}}; \quad \text{RMS velocity of particle} \\
f_{c,s\to p} = 0.5\dfrac{N_s}{dt_s}; \quad \text{frequency of collisions by solvent molecules on particle} \\
f_{c,p\to s} = 0.5\dfrac{N_s}{dt_p}; \quad \text{frequency of collisions by particle on solvent molcules} \\
f_c = f_{c,s\to p} + f_{c,p\to s}; \text{ overall frequency of collisions between solvent and particle} \\
\tau_c = \dfrac{1}{f_c}; \text{ time between solvent/particle collisions}
\end{cases}
$$

$$\begin{cases} N_s \sim 0.886\dfrac{4\pi r_p^2}{\pi r_s^2} = 3.544\left(\dfrac{r_p}{r_s}\right)^2 \\ dt_s = 1\text{Å}\sqrt{\dfrac{m_s}{3k_BT}} \\ dt_p = 1\text{Å}\sqrt{\dfrac{m_p}{3k_BT}} \\ f_c = 0.5\dfrac{N_s}{dt_s} + 0.5\dfrac{N_s}{dt_p} = \dfrac{N_s}{2}\left(\dfrac{1}{dt_s} + \dfrac{1}{dt_p}\right) \\ \tau_c = \dfrac{1}{f_c} \end{cases}$$

$$\begin{cases} f_c = \dfrac{3.544}{2}\left(\dfrac{r_p}{r_s}\right)^2 \dfrac{1}{1\text{Å}}\left(\sqrt{\dfrac{3k_BT}{m_s}} + \sqrt{\dfrac{3k_BT}{m_p}}\right) = 1.772\cdot 10^{10}\left(\dfrac{r_p}{r_s}\right)^2 \sqrt{3k_BT}\left(\dfrac{1}{\sqrt{m_s}} + \dfrac{1}{\sqrt{m_p}}\right) \\ \tau_c = \dfrac{1}{f_c} \end{cases}$$

$$\boldsymbol{\tau_c = \frac{9.775\cdot 10^{-11}}{\sqrt{k_BT}}\left(\frac{r_s}{r_p}\right)^2 \frac{\sqrt{m_s m_p}}{\sqrt{m_s}+\sqrt{m_p}}}$$

Derivation of the expression for Γ_K

$$\begin{cases} \tau_r > 10\tau_c; \quad \textit{condition for Brownian regime} \\ \tau_c = \dfrac{9.775\cdot 10^{-11}}{\sqrt{k_BT}}\left(\dfrac{r_s}{r_p}\right)^2 \dfrac{\sqrt{m_s m_p}}{\sqrt{m_s}+\sqrt{m_p}}; \quad \textit{collisional time} \\ \tau_r = \dfrac{2}{9}\dfrac{\rho_p}{\eta} r_p^2; \quad \textit{relaxation time} \\ v_{RMS,s} = \sqrt{\dfrac{3k_BT}{m_s}}; \quad \textit{RMS velocity of the solvent in 3D} \\ m_p = \dfrac{4}{3}\pi\rho_p r_p^3; \quad \textit{mass of the particle} \end{cases}$$

$$\begin{cases} \frac{2}{9}\frac{\rho_p}{\eta}r_p^2 > \frac{9.775 \cdot 10^{-10}}{\sqrt{k_B T}}\left(\frac{r_s}{r_p}\right)^2 \frac{\sqrt{m_s m_p}}{\sqrt{m_s}+\sqrt{m_p}} \\ \sqrt{m_s} = \frac{\sqrt{3k_B T}}{v_{RMS,s}} \\ \sqrt{m_p} = \frac{\sqrt{3k_B T}}{v_{RMS,p}} \end{cases}$$

$$\rho_p r_p^4 > \frac{4.4899 \cdot 10^{-9}\eta r_s^2}{\sqrt{k_B T}} \frac{\frac{\sqrt{3k_B T}}{v_{RMS,s}}\frac{\sqrt{3k_B T}}{v_{RMS,p}}}{\frac{\sqrt{3k_B T}}{v_{RMS,s}}+\frac{\sqrt{3k_B T}}{v_{RMS,p}}} = 7.7767 \cdot 10^{-9}\eta r_s^2 \frac{\frac{1}{v_{RMS,s}v_{RMS,p}}}{\frac{v_{RMS,s}+v_{RMS,p}}{v_{RMS,s}v_{RMS,p}}} = \frac{7.7767 \cdot 10^{-9}\eta r_s^2}{v_{RMS,s}+v_{RMS,p}}$$

$$\rho_p r_p^4 > \frac{7.7767 \cdot 10^{-9}\eta r_s^2}{v_{RMS,s}+v_{RMS,p}}$$

Which, in the worst case scenario where $v_{RMS,p} = v_{RMS,s}$ would give

$$\rho_p r_p^4 > \frac{3.888 \cdot 10^{-9}\eta r_s^2}{v_{RMS,s}}$$

If we define a parameter $\boldsymbol{\Gamma_K}$ whose value only depends on the solvent and the temperature we can simplify the expression as

$$\rho_p r_p^4 > \Gamma_K$$

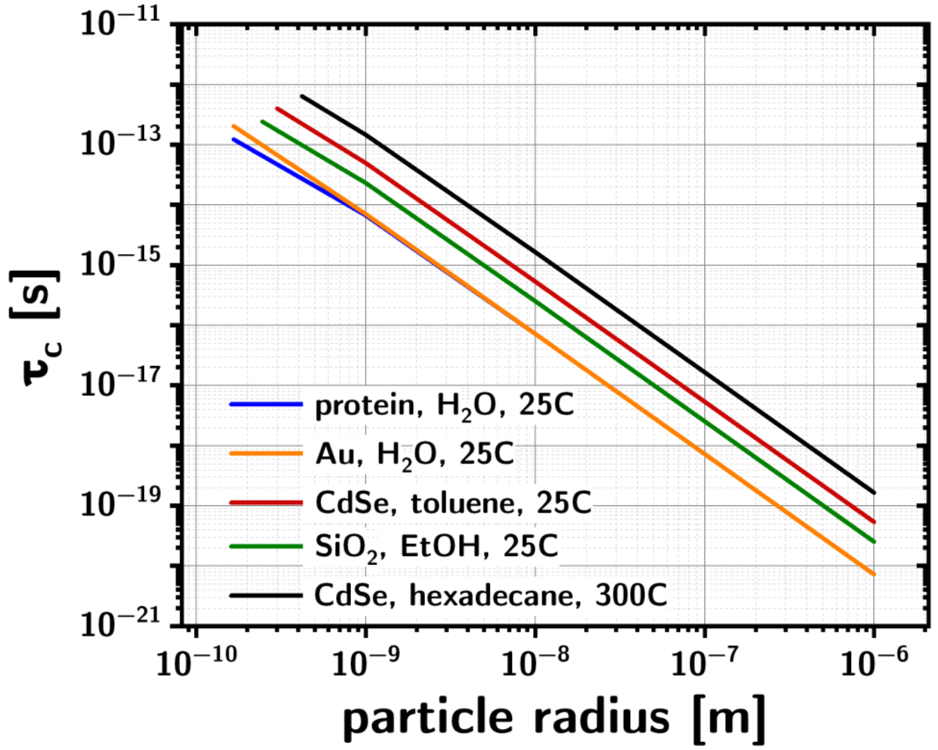

Figure S1. ***Collisional timescale as a function of solvent/particle characteristics and temperature.*** The calculated value of the collisional timescale τ_c (according to Eq. 3) as a function of the particle radius, for a range of solvents, temperatures and particle phases.

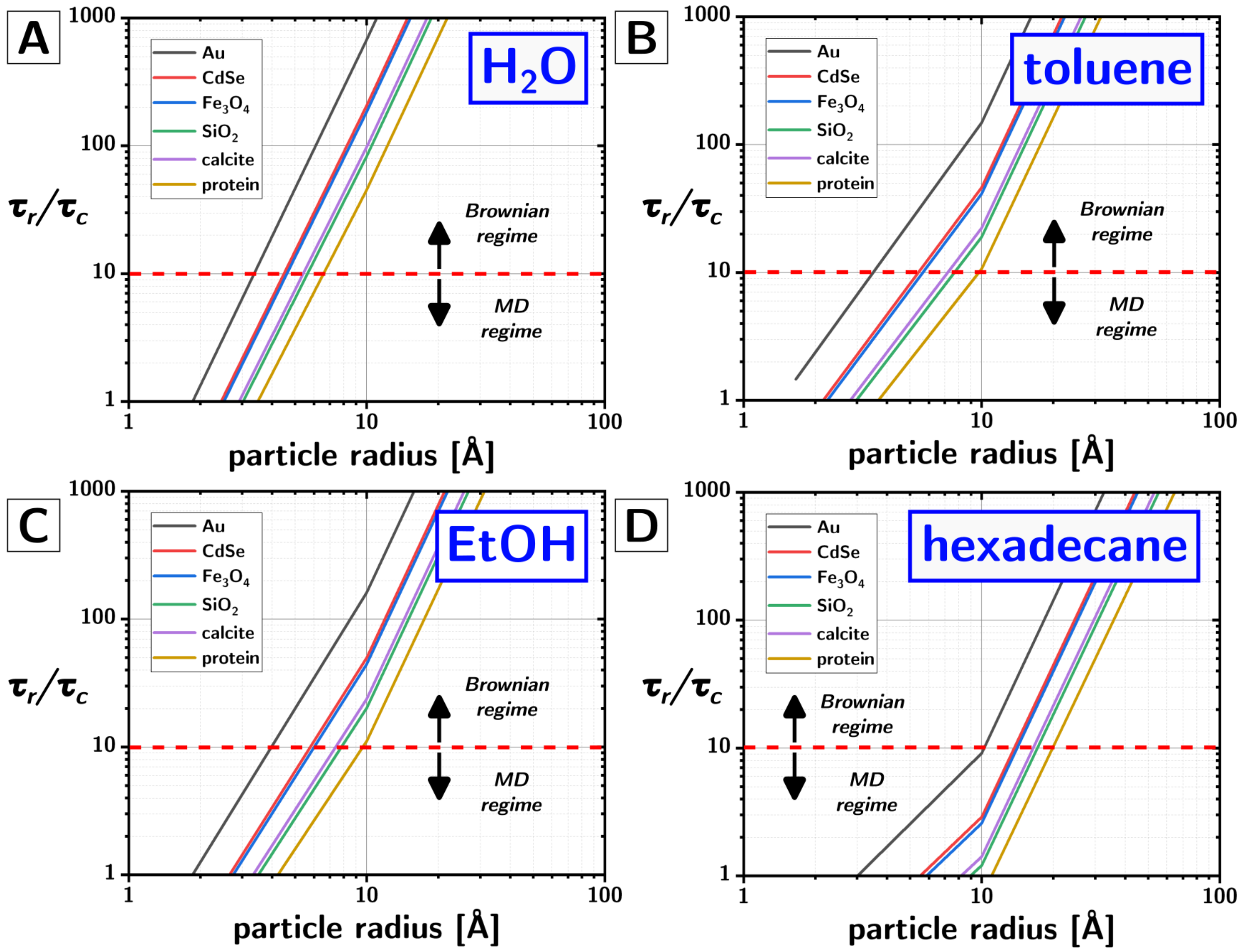

Figure S2. ***Collisional and relaxation timescales as a function of particle radii, particle phases and solvents.*** The graphs overlay, for the same solvent, the ratio between relaxation and collisional time (in essence, the mean number of collisions between solvent molecules and particles that occur during a relaxation time) as a function of the particle radius (for different solid phases). Standard temperatures and pressures are assumed. As discussed in the main text, the Brownian regime can be argued to begin for $\tau_r/\tau_c>10$. The panels show that for most solvents and phases, subnanometric particles can still be described in a Brownian formalism that describes the interactions with the solvent through a diffusivity.

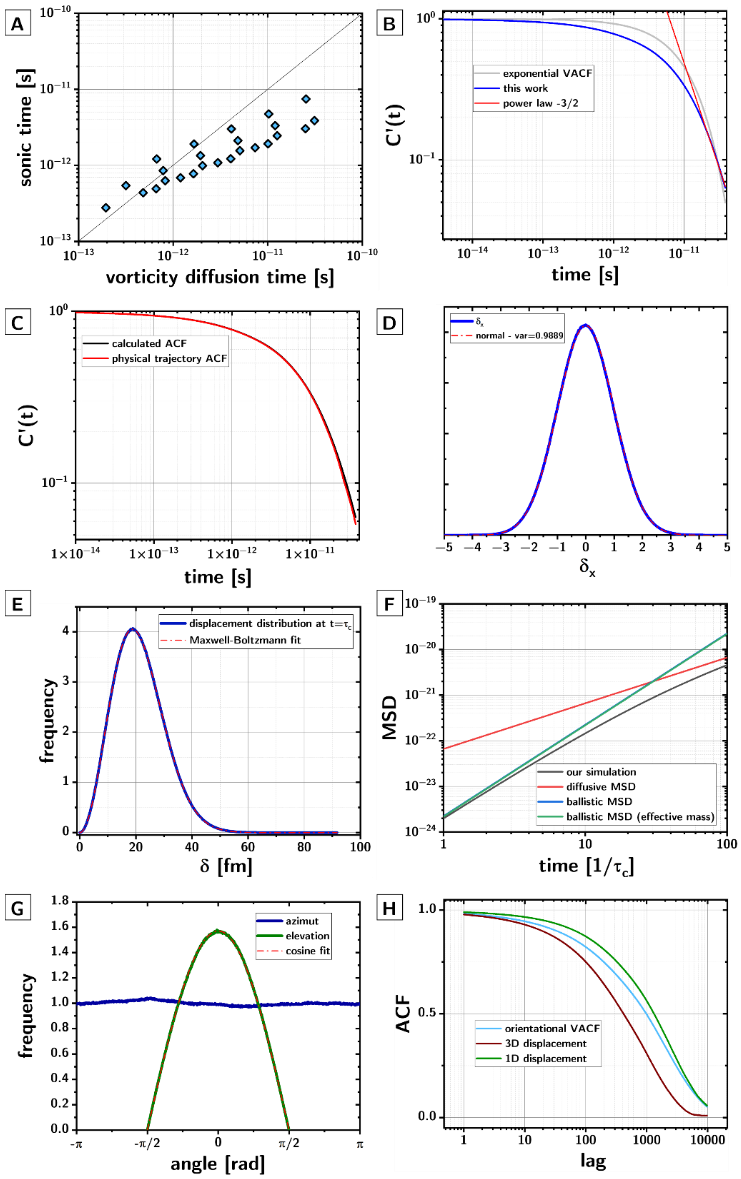

Figure S3. ***Computational diagnostics.*** ***A.*** Relation between sonic time and vorticity diffusion time for the simulation parameters explored. **B.** Comparison between normalized VACFs (exponential in grey vs according to Eq. 5 in blue); the red line shows the algebraic decay fit with a -3/2 exponent. **C.** Comparison between the VACF calculated from Eq. 5 (black) and the one derived from the physical trajectory generated from the β_i coefficients (red). **D.** Distribution of the time series of one-dimensional univariant displacements in the physical trajectory (blue) and the gaussian fit (red dashed). **E.** Distribution of the 3D displacements magnitudes in a physical trajectory (blue) and Maxwell-Boltzmann fit (red dashed). **F.** Comparison between the MSD as a function of time (in τ_c units) from a physical trajectory derived from the VACF in Eq. 5 (black), from a ballistic motion (blue), ballistic motion accounting for the effective mass (green) and from a diffusive motion (red). **G.** Distributions of azimuths and elevations in the displacement orientations in a physical trajectory. The dependence on the angle of both is consistent with a uniform distribution on the spherical angle. **H.**

Comparison of the ACF (at lag=1) calculated from a physical trajectory (orientational in cyan, 3D displacement magnitude in dark red, and 1D displacement magnitude in green)
